# Spectrum of ophthalmic diseases in children hospitalized in a tertiary ophthalmology hospital in China from 2010 to 2019

**DOI:** 10.1186/s12886-022-02533-5

**Published:** 2022-07-19

**Authors:** Xia Zhang, Fan Li, Jiaming Rao, Hao Fang, Wei Zhu

**Affiliations:** 1 State Key Laboratory of Ophthalmology, Zhongshan Ophthalmic Center, Sun Yat-sen University, Guangzhou, 510060 Guangdong Province China; 2 Eye Center, Zhongshan City People’s Hospital, Zhongshan, Guangdong China; 3 Foshan Fetal Medicine Institute, Affiliated Foshan Maternity & Child Healthcare Hospital, Southern Medical University (Foshan Maternity & Child Healthcare Hospital), Foshan, China; 4 Department of Toxicology, Guangzhou Center for Disease Control and Prevention, Guangzhou, 510440 China; 5 Institute of Public Health, Guangzhou Medical University, Guangzhou, 510440 China

**Keywords:** Pediatric, Ophthalmic diseases, Refractive errors, Strabismus, Visual impairment

## Abstract

**Background:**

Describing the pattern of pediatric eye diseases is necessary for appropriate eye care in children. This study explored the spectrum and characteristics of pediatric ophthalmic diseases in a typical tertiary ophthalmology hospital in China.

**Methods:**

A retrospective study was conducted at a tertiary ophthalmology hospital between 2010 and 2019 in Guangzhou, China. This study included 44,552 inpatients who were younger than 18 years old. Demographic and diagnostic data were collected from the electronic medical record system. Multiphase regression analysis was used to estimate trends in the annual percentages of ten common ophthalmic diseases.

**Results:**

From 2010 to 2019, 44,552 inpatients met the inclusion criteria. The majority were male (61.9%), aged 7 to 12 years (30.3%) and self-paying (56.6%). The top ten conditions were refractive error (41.2%), strabismus (36.1%), cataract (13.6%), trauma (11.8%), congenital ptosis (8.8%), tumor (8.1%), amblyopia (7.1%), glaucoma (7.0%), entropion and trichiasis of eyelid (7.0%), and retinal detachment (6.5%). The annual percentage changes (APCs) for refractive error, strabismus, and retinal detachment were 9.3% (95% CI, 8.1–10.5%), 4.7% (95% CI, 3.8–5.6%) and − 2.8% (95% CI, − 5.1% to − 0.4%) respectively. For trauma, the average APC (AAPC = -9.2%, (95% CI, − 12.1% to − 6.2%) decreased gradually from 2010 to 2015 (APC = -4.2% (95% CI, − 8.8-0.7%)) and decreased rapidly from 2015 to 2019 (APC = -15.1% (95% CI, − 21.0% to − 8.7%)).

**Conclusions:**

Pediatric ophthalmic diseases are common in China. Preventive strategies and health education aimed at the prevention of refractive error, strabismus, and entropion and trichiasis of eyelid will be crucial in reducing the burden of pediatric ophthalmic diseases on health care systems and human development.

**Supplementary Information:**

The online version contains supplementary material available at 10.1186/s12886-022-02533-5.

## Background

Vision is one of the most dominant senses and is crucial for children’s physical and mental development. More than three-quarters of early learning experience is acquired via visual stimuli. Hence, visual impairment in early life is harmful to children’s growth and development, as it can lead to delayed language, emotional, social and cognitive development; lower levels of educational achievement; and lifelong consequences [1]. According to data published by World Health Organization (WHO), at least 2.2 billion people have near or distant vision impairment worldwide; of them, 19 million are children, and 1.26 million are bilaterally blind [2, 3]. Children’s characteristics are different than those of adults and can affect ophthalmology visits. Therefore, describing the pattern of pediatric eye diseases is necessary for appropriate eye care in children.

Several studies on the spectrum of pediatric ocular diseases have been conducted [4–7], but data from China are limited. We conducted a hospital-based study in South China to assess the specific patterns and frequencies of pediatric eye diseases from 2010 to 2019 and to investigate the burden and trends of these diseases. The findings will be helpful for pediatric eye care programs and can serve as a basis for the development of a pediatric ophthalmology subspecialty in tertiary hospitals.

## Methods

### Data source

This study was performed at Zhongshan Ophthalmic Center (ZOC), Sun Yat-sen University, which is a leading and internationally renowned eye center located in Guangzhou, China. ZOC provides services to ophthalmic patients throughout the country, as this eye hospital is the first tertiary ophthalmology hospital and possesses advanced medical technology.

### Study design

We conducted a retrospective cross-sectional study. The inclusion criteria were inpatients aged younger than 18 years who were hospitalized at ZOC from 2010 to 2019; only the first recorded hospitalization was included. The exclusion criteria included hospitalization events other than the first event in the same year and foreign nationality, and retinopathy of prematurity (ROP) were not included in our study as these children were not seen in our hospital. Extracted data included the final diagnosis and demographic information. Extracted data included the final diagnosis and demographic information The International Classification of Diseases (ICD) codes used to classify diagnoses are described in Table S1. Patients were divided into four age groups: 0–3 years, 4–6 years, 7–12 years and 13–17 years. The study was approved by the Institutional Ethics Committee of ZOC, Sun Yat-sen University. Because the database does not contain any direct patient identifiers, the need for signed informed consent was waived by Zhongshan ophthalmic center ethics committee. All methods were performed in accordance with relevant guidelines and regulations.

### Data analysis

Data were stored and analyzed in SPSS version 24.0 (SPSS Inc., Chicago, IL, USA) and Joinpoint Regression Program version 4.5 (Statistical Research and Applications Branch, National Cancer Institute, Bethesda, MD). Figures were constructed in GraphPad Prism version 5.0 and simple frequencies or cross-tabulations and bar or line graphs are presented. Descriptive statistics were calculated for patient demographics (age, sex, cost, primary payer, region, nationality) and diagnoses (cataract, glaucoma, refractive error, strabismus, amblyopia, and so on). The chi-squared test was used to compare differences between groups. Multiphase regression analysis was used to estimate the trends of the annual percentage. The annual percentage change (APC), along with the corresponding 95% confidence interval (95% CI), was calculated for each trend. The APC values that were statistically significant indicated temporal changes in the percentage trend over the study period. All *p* values were two-tailed, and a *p* value less than 0.05 was considered statistically significant.

## Results

### Demographics

The mean (standard error) age of all inpatient children was 7.2 (4.7) years, with those aged 7–12 years comprising 30.3% of the study population. Almost all pediatric inpatients were of Han ethnicity (97.7%), male (61.9%) and self-paying (56.6%). The largest number of visits was observed among patients from south China (68.4%). Table 1 shows the demographics of all the inpatients by age.

**Table 1:** Demographic information of pediatric patients with eye disease presenting to ophthalmology hospital in the Guangzhou, 2010–2019

Characteristic	0–3 Yrs *N* = 10,779(24.2%)	4–6 Yrs *N* = 12,523(28.1%)	7–12 Yrs *N* = 13,493(30.3%)	13–17 Yrs *N* = 7757(17.4%)	Total *N* = 44,552(100%)
Sex					
Male	6547(60.7)	7506(59.9)	8416(62.4)	5093(65.7)	27,562(61.9)
Female	4232(39.3)	5017(40.1)	5077(37.6)	2664(34.3)	16,990(38.1)
Insurance^a^					
Public	4279(39.7)	5179(41.4)	5484(40.6)	2642(34.1)	17,584(39.5)
Commercial	16(0.1)	33(0.3)	33(0.2)	16(0.2)	98(0.2)
Self-pay	6052(56.1)	6909(55.2)	7477(55.4)	4784(61.7)	25,222(56.6)
Uninsured	432(4)	402(3.2)	499(3.7)	315(4.1)	1648(3.7)
Region					
North	51(0.5)	38(0.3)	42(0.3)	24(0.3)	155(0.3)
Northeast	22(0.2)	46(0.4)	29(0.2)	21(0.3)	118(0.3)
East	1351(12.5)	1505(12.0)	1734(12.9)	890(11.5)	5480(12.3)
South-central	1367(12.7)	1592(12.7)	1722(12.8)	852(11.0)	5533(12.4)
South	7246(67.2)	8621(68.8)	9129(67.7)	5484(70.7)	30,480(68.4)
Southwest	666(6.2)	629(5.0)	752(5.6)	433(5.6)	2480(5.6)
Northwest	76(0.7)	92(0.7)	85(0.6)	53(0.7)	306(0.7)
Ethnicity					
Han	10,539(97.8)	12,261(97.9)	13,205(97.9)	7536(97.2)	43,541(97.7)
Others	240(2.2)	262(2.1)	288(2.1)	221(2.8)	1011(2.3)

^a^Public insurance includes Basic medical insurance for nonworking urban residents, Basic medical insurance for urban workers, new rural cooperative medical insurance and other government programs; uninsured includes patients with no charge or not known

### Morbidity and epidemic trends

From 2010 to 2019, 44,552 children were hospitalized in the eye clinic, of whom 38,425 (58.6%) patients were first-time visitors. Refractive error was one of the most common disorders (41.2%), followed by strabismus (36.1%) and cataract (13.6%). Cataract (23.3%), tumor (19.6%), and glaucoma (11.7%) accounted for the highest morbidity in 0–3 years. The percentage of congenital ptosis (13.2%), amblyopia (9.7%) and entropion and trichiasis of eyelid (10.3%) were highest in 4–6 years. The percentages of strabismus (48.6%) and trauma (13.2%) were highest in 7–12 years, and the percentages of refractive error (56.5%) and retinal detachment (16.8%) were highest in 13–17 years. All these differences were statistically significant (*P* < 0.001). Table 2 shows the percentages of the top ten pediatric eye diseases among all inpatient children by age group.

**Table 2:** Distribution of the top ten pediatric eye disease by age group

Diagnoses	0–3 Yrs *N* = 10,779(24.2%)	4–6 Yrs *N* = 12,523(28.1%)	7–12 Yrs *N* = 13,493(30.3%)	13–17 Yrs *N* = 7757(17.4%)	Total *N* = 44,552 (100%)	*p*
Refractive error	1187(11.0)	5722(45.7)	7062(52.3)	4379(56.5)	18,350(41.2)	0.000
Strabismus	1435(13.3)	5254(42.0)	6561(48.6)	2846(36.7)	16,096(36.1)	0.000
Cataract	2509(23.3)	1249(10.0)	1335(9.9)	951(12.3)	6044(13.6)	0.000
Trauma	1093(10.1%)	1549(12.4%)	1776(13.2%)	852(11.0%)	5270(11.8%)	0.000
Congenital ptosis	1089(10.1)	1651(13.2)	776(5.8)	388(5.0)	3904(8.8)	0.000
Tumors	2117(19.6)	638(5.1)	537(4.0)	331(4.3)	3623(8.1)	0.000
Amblyopia	133(1.2)	1210(9.7)	1180(8.7)	651(8.4)	3174(7.1)	0.000
Glaucoma	1265(11.7)	483(3.9)	736(5.5)	634(8.2)	3118(7.0)	0.000
Entropion and trichiasis of eyelid	784(7.3)	1290(10.3)	800(5.9)	223(2.9)	3097(7.0)	0.000
Retinal detachment	324(3.0)	365(2.9)	911(6.8)	1300(16.8)	2900(6.5)	0.000

**Fig. 1:**
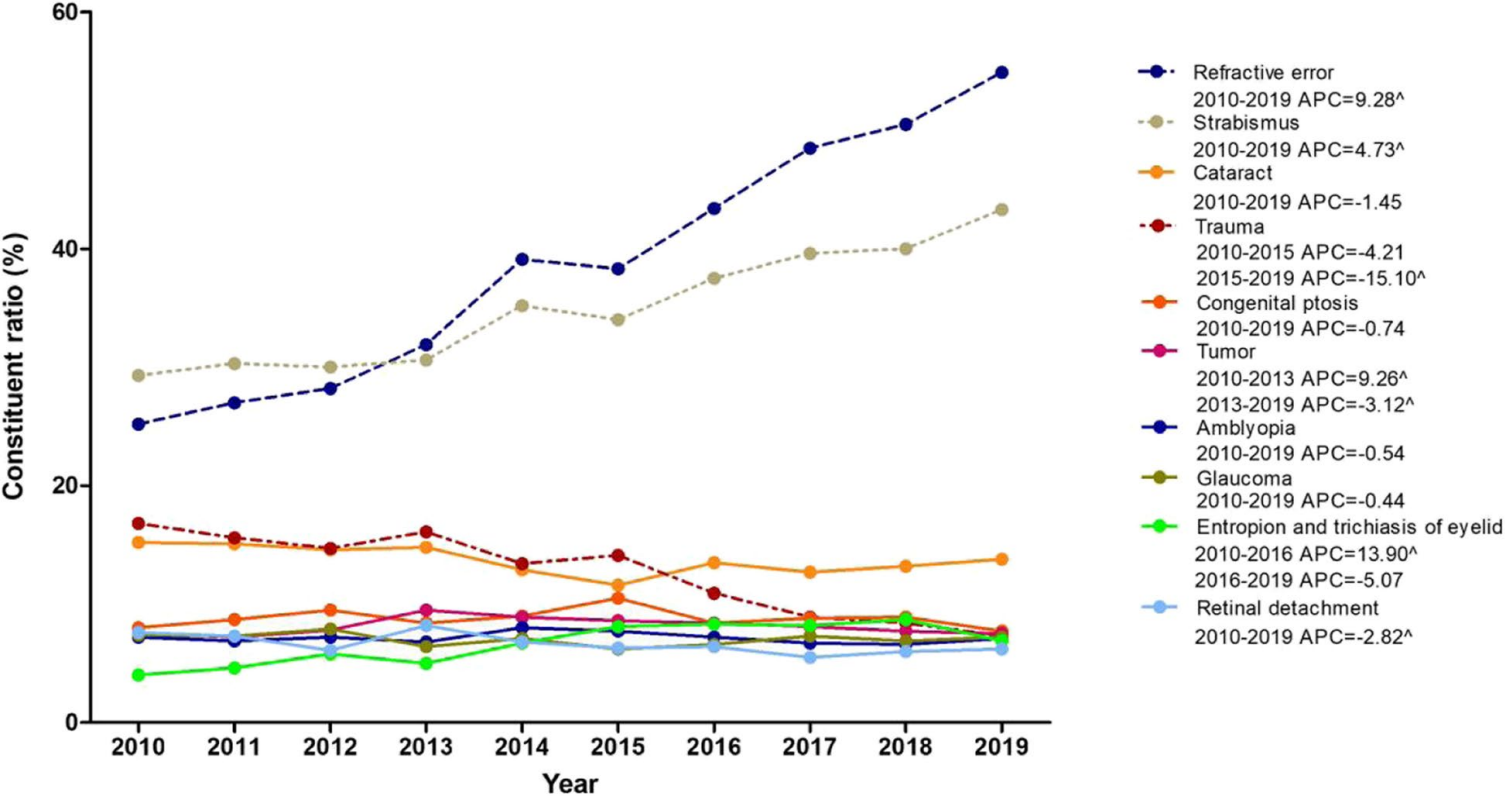
Trends of the top ten eye diseases among pediatric inpatients a ZOC from 2010 to 2019

The percentage of refractive error increased approximately 1.2-fold from 25.2% in 2010 to 54.9% in 2019, with an increase of 9.3% per year (95% CI, 8.1–10.5%). The percentage of strabismus increased from 29.3% in 2010 to 43.3% in 2019, with an APC of 4.7% (95% CI, 3.8–5.6%). The percentage of retinal detachment decreased from 7.6% in 2010 to 6.2% in 2019, with an APC of − 2.8% (95% CI, − 5.1% to − 0.4%). The percentage of trauma decreased from 16.8 to 7.3% during the study period; it initially decreased slowly from 2010 to 2015, with an APC of − 4.2% (95% CI, − 8.8-0.7%), then decreased rapidly from 2015 to 2019, with an APC of − 15.1% (95% CI, − 21.0% to − 8.7%) and an AAPC of − 9.2% (95% CI, − 12.1% to − 6.2%). The largest change in ophthalmic diseases was observed in children aged 7–12 years, followed by aged 4–6 years. Figure 1 shows detailed information about trends of pediatric eye diseases.

### Trauma and causes

Among the pediatric inpatients with trauma, 74.7% were male (*P* < 0.001), and the percentage was highest in 7–12 Yrs (33.7%). Regarding the type of injury, the majority were penetrating wounds in all age groups, with a total percentage of 67.8%. Regarding the injured structure of the eye, the lens (50.9%) was the most affected. Table 3 describes the distribution of ocular injuries by age group.

**Table 3:** Distribution of ocular injuries by age group

Groups	0–3 Yrs *N* = 1093(20.7%)	4–6 Yrs *N* = 1549(29.4%)	7–12 Yrs *N* = 1776(33.7%)	13–17 Yrs *N* = 852(16.2%)	Total *N* = 5270 (100%)	*p*
Type						
Penetrating wound	855(78.2)	1124(72.6)	1116(62.8)	477(56.0)	3572(67.8)	0.000
Contusion	198(18.1)	217(14.0)	326(18.4)	176(20.7)	917(17.4)	0.000
Burn or Corrosion	59(5.4)	75(4.8)	81(4.6)	24(2.8)	239(4.5)	0.083
Superficial injury	39(3.6)	54(3.5)	60(3.4)	25(2.9)	178(3.4)	0.950
Tissue						
Lens	518(47.4)	845(54.6)	936(52.7)	385(45.2)	2684(50.9)	0.000
Retina	99(9.1)	115(7.4)	170(9.6)	130(15.3)	514(9.8)	0.000
Orbit	11(1.0)	27(1.7)	86(4.8)	91(10.7)	215(4.1)	
Optic nerve	12(1.1)	14(0.9)	65(3.7)	67(7.9)	158(3.0)	0.000
Glaucoma	12(1.1)	14(0.9)	51(2.9)	28(3.3)	105(2.0)	0.000

**Fig. 2:**
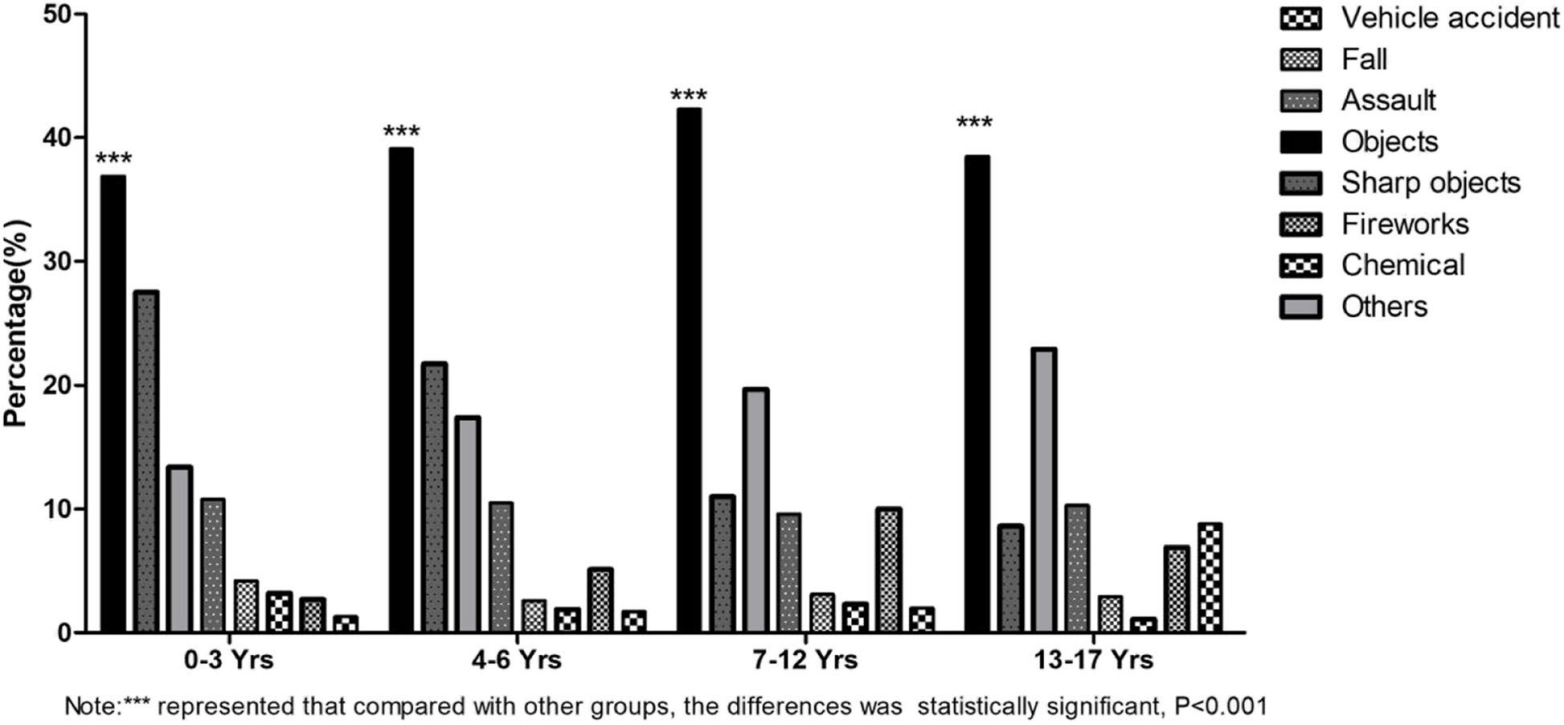
Frequency of injury mechanisms in pediatric inpatients for ocular trauma in ZOC from 2010 to 2019

Excluding the classification “others”, the most common source of trauma was objects (39.6%) in.

all age groups, followed by sharp objects (17.2%), except in the 13–17 years group, for whom the second most common source was assault (10.3%). Figure 2 shows the frequencies of trauma causes classified by age. Furthermore, the number of injuries caused by vehicle accidents more than doubled over the study period (from 2.3 to 5.1%, *P* < 0.000), and the number of injuries caused by falls increased more than tenfold (from 0.9 to 6.9%, *P* < 0.001).

### Congenital disease

The percentage of congenital ophthalmic diseases was 25.7% in our study. Regarding diseased tissues, the most common was the ocular adnexa (41.7%), followed by the lens (34.9%). Table 4 shows the patterns and frequencies of congenital ophthalmic diseases by age group.

**Table 4:** Distribution of congenital ophthalmic diseases by age group

Types	0–3 Yrs *N* = 4867(42.4%)	4–6 Yrs *N* = 3353(29.2%)	7–12 Yrs *N* = 2134(18.6%)	13–17 Yrs *N* = 1117(9.7%)	Total *N* = 11,471(100%)	*p*
Ocular Adnexa^a^	1418(29.1)	1951(58.2)	945(44.3)	467(41.8)	4781(41.7)	0.000
Lens^b^	2214(45.5)	906(27.0)	617(28.9)	271(24.3)	4008(34.9)	0.000
Glaucoma^c^	924(19.0)	217(6.5)	269(12.6)	193(17.3)	1603(14.0)	0.000
Eyeball^d^	67(1.4)	45(1.3)	14(0.7)	15(1.3)	141(1.2)	0.068

Note:^a^The code for ocular adnexa injury is Q10, which includes Q10.0: congenital ptosis, Q10.1: congenital ectropion, Q10.2: congenital entropion, Q10.3: other congenital malformation of the eyelid. ^b^The code for congenital lens abnormalities is Q12, which includes Q12.0: congenital cataract, Q12.1: congenital displaced lens, Q12.2: coloboma of lens, Q12.3: congenital aphakia, Q12.4: spherophakia, Q12.8: other congenital lens malformations and Q12.9 congenital lens malformation, unspecified. ^c^The code for congenital glaucoma is Q15.0; ^d^The code for congenital malformation of the eyeball is Q11. See details in supplementary Table 1

### Tumors and infection

**Fig. 3:**
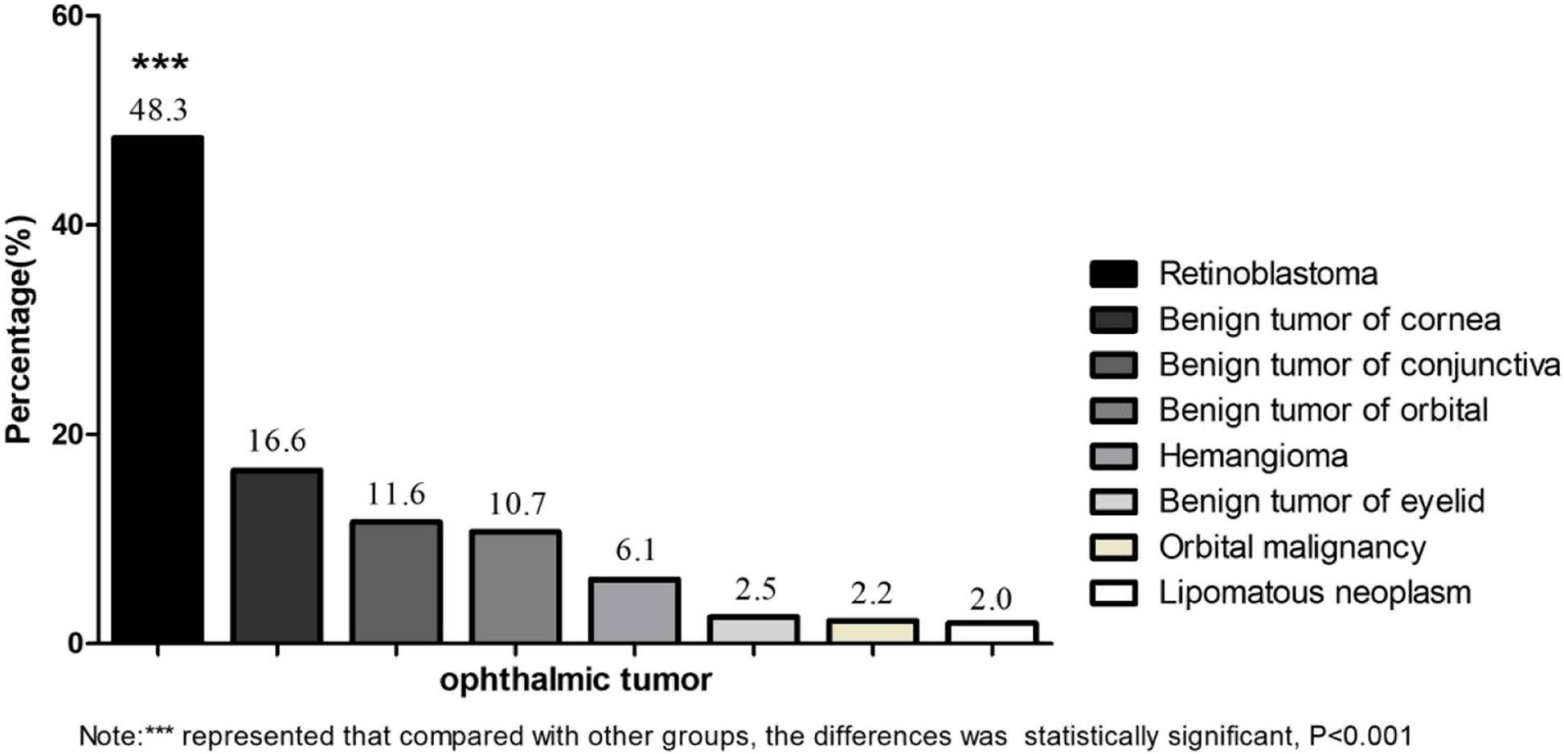
Frequency of ophthalmic tumors in children at ZOC from 2010 to 2019

Almost half of the tumors were retinoblastomas (48.3%), followed by benign tumors of the cornea (11.6%) (*P* < 0.001), and the prevalence was highest in those aged 0–3 years (19.4%). Regarding eye infections, the top three were keratitis (38.0%), endophthalmitis (24.4%) and iridocyclitis (13.8%). The percentage was highest in those aged 7–12 years(33.0%), and the difference was statistically significant (*P* < 0.001). Table S2 and Fig. 3 show the patterns and frequencies of ophthalmic tumors, and Table 5 shows the patterns and frequencies of infections by age group.

**Table 5:** Distribution of ocular inflammation by age group

Diagnoses	0–3 Yrs *N* = 549(20.2%)	4–6 Yrs *N* = 850(31.3%)	7–12 Yrs *N* = 894(33.0%)	13–17 Yrs *N* = 419(15.4%)	Total *N* = 2712(100%)	*p*
Keratitis	201(36.6)	393(46.2)	312(34.9)	125(29.8)	1031(38.0)	0.000
Endophthalmitis	128(23.3)	232(27.3)	226(25.3)	75(17.9)	661(24.4)	0.003
Iridocyclitis	42(7.7)	75(8.8)	155(17.3)	101(24.1)	373(13.8)	0.000
Hordeolum	131(23.9)	77(9.1)	50(5.6)	15(3.6)	273(10.1)	0.000
Conjunctivitis	20(3.6)	45(5.3)	64(7.2)	24(5.7)	153(5.6)	0.042
Orbital infection	24(4.4)	15(1.8)	46(5.1)	27(6.4)	112(4.1)	0.000
Dacryoadenitis	20(3.6)	22(2.6)	30(3.4)	36(8.6)	108(4.0)	0.000
Choroiditis	1(0.2)	8(0.9)	6(0.7)	10(2.4)	25(0.9)	0.003

## Discussion

In this study, we analyzed the medical records data of 44,552 pediatric patients with eye diseases in China from 2010 to 2019. We studied all diagnoses, including principal diagnosis and secondary diagnosis, and found that during this period, refractive error was the most common disorder, followed by strabismus and cataract. The percentage of refractive error and strabismus increased, whereas the percentage of trauma and retinal detachment decreased.

Vision is the most dominant of the senses and is vital in every aspect of our lives. Regarding ocular diseases, children have a unique situation because they are unable to articulate or express their experiences [8]. The uncorrected refractive error remains a leading cause of vision impairment in all countries among children [9]. In our study, refractive error includes hypermetropia, myopia, astigmatism, anisometropia and aniseikonia. We found that the percentage of refractive error increased by approximately 1.2-fold from 2010 to 2019, and refractive error was most likely to occur in those aged 13–17 years, similar to the results of Zhao’s study [10]. Although genetic factors influence refractive error, a recent review demonstrated that limited outdoor activity and increased electronic device use are key environmental risk factors for myopia [11]. Furthermore, the increased percentage of refractive error might also be associated with air pollution and artificial light pollution. Some studies have indicated that exposure to ambient air pollution, such as PM_2.5_ and NOx, promotes myopia [12]. Artificial light pollution contributes to disordered circadian rhythms and may influence refractive development and myopia [13]. Strabismus was the second most common disease observed in this study. Similar to a previous study, there was a higher percentage among older children than among younger children [14]. If strabismus is not treated in a timely manner in children, it may have a dramatic impact on their daily life activity and learning ability and may impair their physiological and psychological performance [15]. Refractive error and strabismus are closely related [16, 17]. The higher percentage reported in older children might be due to older children having better expressive ability, making it easier to detect visual problems. Regular screening for refractive error and refractive services can prevent poor performance in school and the development of amblyopia and strabismus. Many interventions, such as vision spectacle lenses, orthokeratology lenses, and low-dose atropine, can reduce refractive error progression. A network analysis indicated that two or more interventions can significantly reduce the progression of myopia when compared with single-vision spectacle lenses or a placebo [18]. Refractive error should be detected and treated early, and the treatment may need to include multiple interventions.

Ocular trauma was the fourth most common eye disease observed in this study, similar to a previous study [19] and was more likely to occur in those aged 7–12 years. The most common cause was force by an object, followed by assault in those aged 13–17 years. Every year, serious ocular trauma affects approximately 6 million children [20]. Wounded children live with permanent visual defects, which inevitably influence their physical and psychological health. Ocular trauma also imposes a significant socioeconomic burden on families [21]. In our study, ocular trauma cause with the highest proportion was penetrating wounds, as observed in a previous study [22, 23]. Even though ocular trauma showed a decreasing trend, it is still a serious worldwide public health problem and can be prevented in 90% of cases [24–26]. It is important for guardians to repeatedly warn children to avoid risky objects (stones, glass, scissors, fireworks), avoid fights with friends, and wear protective equipment when performing risky activities. One study indicated that wearing protective goggles during high-risk activities can effectively prevent ocular trauma [27]. It is necessary for adults to properly store dangerous items around the house [28] and to adequately supervise playing children.

In this study, congenital ptosis, the first congenital ophthalmopathy to be discovered, was the fifth most common disease and accounted for 81.7% of ocular adnexal diseases. Congenital ptosis is a relatively rare condition compared with the other three congenital oculopathies; it is characterized by abnormal drooping of the upper eyelid that is present at birth or occurs in the first year of life [29]. The prevalence of congenital ptosis in the general population is 0.18–1.41% [30, 31]; in our study, the percentage was 8.8%. The etiology of congenital ptosis includes autosomal inheritance and systemic syndromes. It may account for both anomalies of extraocular muscle development and innervation [32]. If ptosis is not treated, it may lead to abnormal visual development, resulting in amblyopia, strabismus and refractive error [33]. Children with congenital ptosis should be examined regularly to monitor their visual development, and early surgery should be conducted to prevent psychological impacts on children and in severe cases of monocular ptotic eye to avoid sensory deprivation amblyopia with the aid of proper refractive correction..The higher incidence of congenital ptosis in our study may be due to our center being the Number 1 eye hospital in the country and its high-level operation ability. Ultimately, the most common and resolutive approach for congenital ptosis is surgical intervention.

Although retinoblastoma is a rare malignancy of the eye, it is the most common intraocular malignancy in children and occurs most frequently in those aged 4 years or younger [34]. In this study, almost half of the tumors were retinoblastomas (48.3%), and the prevalence was highest in children aged 0 to 3 years. Worldwide, most retinoblastoma cases occur in Asia (43–53%) [35, 36], probably because retinoblastoma is a prototypical genetic cancer [37]. The priority goal of retinoblastoma management is to save the child’s life; secondary goals are globe salvage and optimization of visual function [38]. Chemotherapy is a potential vision-keeping approach, but enucleation must be implemented promptly for advanced retinoblastoma [34]. Retinoblastoma is considered a curable malignant tumor with a near 100% survival rate if timely intervention is conducted during the early stage at initial presentation [39]. Early diagnosis and timely intervention are key for improving survival in children with retinoblastoma. Therefore, it is necessary to strengthen knowledge and education about retinoblastoma among primary care providers and parents to promote early diagnosis and timely intervention. Furthermore, universal screening and highly subsidized or free treatment are necessary for patients with a low socioeconomic status.

Bacteria are associated with many types of ocular infections, such as conjunctivitis, keratitis, and endophthalmitis [40]. In this study, the most common eye infection in children was keratitis (38.0%), followed by endophthalmitis (24.4%). Keratitis is one of the most serious eye infections and may progress to endophthalmitis [41]. Pediatric endophthalmitis is a rare but devastating condition, and the rate of endophthalmitis after cataract surgery ranges from 0.1 to 0.45% [42, 43], while the rate after eye trauma ranges from 2.8 to 71.8%, with a relatively high incidence in developing countries [44]. Knowledge of the specific etiology is critical for the effective management of eye infections. A previous study conducted in ZOC showed that trauma was the main etiology of pediatric endophthalmitis and that Streptococcus was the most prevalent organism [45]. Both keratitis and endophthalmitis are potentially devastating ocular infections if not diagnosed and treated early. Therefore, it is important to strengthen research on the etiology of eye infections and provide specific information to doctors and policy-makers to address eye infections. In addition, it is crucial to strengthen health education, when eye infection occurs, medical attention should be sought immediately.

Although our study demonstrated several important findings in a cohort recruited from a nationally recognized ophthalmology hospital, there are some limitations to our study. First, this study was hospital-based rather than population-based and retrospective in nature. The inpatient disease prevalence is not representative of the prevalence of such diseases in the population. However, our center is the most authoritative ophthalmic hospital in China, and patients come from all over the country. We included ICD codes for all diagnoses, which helped reduce selection bias. Second, the disease data relies on accurate diagnosis and ICD codes, which depend heavily on the abilities of both physicians and coders; therefore, missing data and errors are possible. Fortunately, in this study, the physicians and coders were professional and responsible. Third, the database does not contain detailed clinical and socioeconomic information, and we could no analyze factors that potentially influenced hospitalization and disease prognosis. Fourth, this study did not report some common ocular morbidities associated with diseases that are commonly treated in the neonatal departments of maternal and child health hospitals or general hospitals in China, such as cerebral visual impairment and retinopathy of prematurity. Finally, the database contains encounter-level data rather than patient-level data, meaning that it is possible that patients who were hospitalized more than once artificially inflated the total number. Nevertheless, we included only the first hospitalization if the patient presented more than once per year. Moreover, we provide valid data from a relatively large sample to describe the disease distribution.

## Conclusions

In conclusion, our study demonstrates that refractive error, strabismus, cataract, trauma, congenital ptosis, tumor, amblyopia, glaucoma, entropion and trichiasis of eyelid, and retinal detachment were the top ten eye diseases in our center. The percentage of eye diseases undergoing great changes, such as refractive error and strabismus increased, while the percentage of retinal detachment and trauma decreased, especially in children aged 7–12 years. It is imperative for guardians to focus efforts on preventing children from accessing risk factors to prevent various conditions that can affect visual development. Vision impairment in children will last a lifetime and seriously affect physical and mental health. Hence, early prevention, early detection, and early treatment are necessary to reduce the incidence of eye diseases in children. Furthermore, appropriate health education, more pediatric ophthalmologists, and regular vision screening are crucial.

## Supplementary Information

**Additional file 1.**

## Data Availability

The all data used to support the findings of this study are available from the corresponding author upon request.

## Abbreviations

APC: Annual percentage change
AAPC: Average annual percentage change
Yrs: Years
CI: Confidence interval
ZOC: Zhongshan Ophthalmic Center
ICD: International Classification of Diseases
