# Supplementary material for: Spectrum of ophthalmic diseases in children hospitalized in a tertiary ophthalmology hospital in China from 2010 to 2019
Source: BMC Ophthalmol. 2022 Jul 19;22:314. doi: 10.1186/s12886-022-02533-5 (PMC9297649; doi:10.1186/s12886-022-02533-5)
Supplement: Supplementary file 1 — Additional file 1. [file 12886_2022_2533_MOESM1_ESM.docx]

**Title page**

**Title:** Spectrum of ophthalmic diseases in children hospitalized in a tertiary ophthalmology hospital in China from 2010 to 2019

**Authors:** Xia Zhang^1^ Fan Li^1,2^ Jiaming Rao^3^ Hao Fang^1^ Wei Zhu^4,5^

| Table S1 International Classification of Disease (ICD) codes | |
| --- | --- |
| Diagnosis | ICD-10 |
| Glaucoma | H40-H42（except H40.3）,Q15.0,P15.3 |
| Cataract | H26（except H26.1）,Q12.0 |
| Strabismus | H49-H51 |
| Amblyopia | H53.0 |
| Refractive error | H52 |
| Traumas | H26.1, S00.1, S00.2, S01.1, S04.0, S05, T15, T26, H33.302#, H33.503#, H40.3 |
| Superficial injury | S00.2, S05.0, T15 |
| Contusion | S00.1, S05.1 |
| Fracture orbit | S02.1, S02.3, S02.8 |
| Open injury of ocular adnexal | S01.1, S05.4, S05.900# |
| Open injury of eyeball | S05.2, S05.3, S05.5, S05.6, S05.7 |
| Lens trauma | H26.1, S05.805#, S05.806# |
| Traumatic glaucoma | H40.3 |
| retinal trauma | H33.302#, H33.503# |
| Optic neuropathy | S04.00 |
| Burns and corrosions | T26 |
| Tumour | C00-D48 |
| Retinoblastoma | C69.2 |
| Orbital malignancy | C69.6 |
| Lipomatous neoplasm | D17.0 |
| Hemangioma | D18 |
| Benign tumor of eyelid | D23 |
| Benign tumor of cornea | D31.1 |
| Benign tumor of conjunctiva | D31.0 |
| Benign tumor of orbital | D31.6 |
| Amotio retinae | H33.0, H33.2, H33.4, H33.5 |
| Congenital malformation | Q10-Q15 |
| Congenital lens malformation | Q12 |
| Congenital glaucoma | Q15.0 |
| Congenital malformation of eyeball | Q11 |
| Congenital malformation of ocular adnexal | Q10 |
| Hordeolum | H00, H01, H03* |
| Entropion | H02.0 |
| Dacryoadenitis | H04.0, H04.3, H04.4 |
| Orbital infection | H05.0, H05.1, H06.1 |
| Conjunctivitis | H10, H13.0, H13.1, H13.2, H13.3, A71 |
| Keratitis | H16, H19.1*, H19.2*, H19.3* |
| Iridocyclitis | H20, H22.0*, H22.1* |
| Choroiditis | H30, H32.0* |
| Endoophthalmitis | H44.0,H44.1, H45.1* |
| Notice:# represent Guangdong Province ICD extended code library | |

| Table S2 Distribution of the ocular tumor across age group | | | | | |
| --- | --- | --- | --- | --- | --- |
| Diogenes | 0-3 Yrs N=10779(24.2%) | 4-6 Yrs N=12523(28.1%) | 7-12 Yrs N=13493(30.3%) | 13-17 Yrs N=7757(17.4%) | Total N=44552 (100%) |
| Retinoblastoma | 1366(12.7%) | 249(2.0%) | 60(0.4%) | 6(0.1%) | 1681(3.8%) |
| Orbital malignancy | 29(0.3%) | 15(0.1%) | 20(0.1%) | 12(0.2%) | 76(0.2%) |
| Lipomatous neoplasm | 14(0.1%) | 14(0.1%) | 31(0.2%) | 10(0.1%) | 69(0.2%) |
| Hemangioma | 68(0.6%) | 34(0.3%) | 58(0.4%) | 53(0.7%) | 213(0.5%) |
| Benign tumor of eyelid | 26(0.2%) | 22(0.2%) | 24(0.2%) | 16(0.2%) | 88(0.2%) |
| Benign tumor of cornea | 328(3.0%) | 124(1.0%) | 83(0.6%) | 41(0.5%) | 576(1.3%) |
| Benign tumor of conjunctiva | 82(0.8%) | 80(0.6%) | 146(1.1%) | 96(1.2%) | 404(0.9%) |
| Benign tumor of orbital | 181(1.7%) | 69(0.6%) | 56(0.4%) | 65(0.8%) | 371(0.8%) |
| Total | 2094（19.4%） | 607（4.8%） | 478（3.5%） | 299（3.9%） | 3478（7.8%） |
